# Concept learning and the use of three common psychophysical paradigms in the archerfish (*Toxotes chatareus*)

**DOI:** 10.3389/fncir.2014.00039

**Published:** 2014-04-24

**Authors:** Cait Newport, Guy Wallis, Ulrike E. Siebeck

**Affiliations:** ^1^Laboratory for Visual Neuroethology, School of Biomedical Sciences, The University of QueenslandBrisbane, QLD, Australia; ^2^Centre for Sensorimotor Neuroscience, School of Human Movement Studies, The University of QueenslandBrisbane, QLD, Australia; ^3^Queensland Brain Institute, The University of QueenslandBrisbane, QLD, Australia

**Keywords:** visual discrimination, behavior, matched-to-sample, alternative forced-choice, odd-one-out

## Abstract

Archerfish are well known for their specialized hunting technique of spitting water at prey located above the water line. This unique ability has made them a popular focus of study as researchers try to understand the mechanisms involved in targeting and spitting. In more recent years, archerfish have also become an increasingly popular model for studying visual discrimination and learning in general. Until now, only the alternative forced-choice (AFC) task has been used with archerfish, however, they may be capable of learning other classical discrimination tasks. As well as providing alternative, and potentially more efficient, means for testing their visual capabilities, these other tasks may also provide deeper insight into the extent to which an organism with no cortex can grasp the concepts underlying these tasks. In this paper, we consider both the matched-to-sample (MTS) and the odd-one-out (OOO) tasks as they require the subject to learn relatively sophisticated concepts rather than a straight, stimulus-reward relationship, of the kind underlying AFC tasks. A variety of line drawings displayed on a monitor were used as stimuli. We first determined if archerfish could complete the MTS and OOO test and then evaluated their ability to be retrained to new stimuli using a 4-AFC test. We found that archerfish were unable to learn the MTS and had only a limited capacity for learning the OOO task. We conclude that the MTS and OOO are impractical as paradigms for behavioral experiments with archerfish. However, the archerfish could rapidly learn to complete an AFC test and select the conditioned stimulus with a high degree of accuracy when faced with four stimuli, making this a powerful test for behavioral studies testing visual discrimination. In addition, the fish were able to learn the concept of oddity under particular training circumstances. This paper adds to the growing evidence that animals without a cortex are capable of learning some higher order concepts.

## INTRODUCTION

For many organisms, vision represents the primary source of sensory information for guiding behavior. However, to date, the majority of what we have learnt about the processing of visual information has been gleaned through the study of a remarkably small range of higher vertebrates (cat, rabbit, monkey, and human). Because these animals all possess a cerebral cortex, many visual tasks, including object recognition, have been investigated in the context of the considerable processing capacity which a cortex provides, permitting the development of complex and or highly specialized models of how we solve specific visual recognition tasks. However, there is evidence to suggest that much simpler models may be sufficient to explain certain visual recognition abilities. One way to understand more about the mechanisms underlying visual recognition is to determine how animals lacking a cortex process complex visual information. If, for example, animals without a cortex are able to perform specific tasks competently, it suggests that, in that instance at least, specialized cortical systems may well not be required after all. Conversely, if they struggle to perform a task this may indicate a significant processing contribution of the cortex in that case. Fish represent an ideal model organism as they lack a cortex, yet show sophisticated visual behaviors and can be trained to complete behavioral experiments.

The majority of our knowledge about the visual system of fish comes from the fields of morphology and electrophysiology, with only relative few studies choosing to employ behavioral experiments to explore the animal’s visual abilities. Psychophysical (behavioral) tests offer an important means for determining properties of the visual capabilities of fish (e.g., absolute sensitivity, contrast sensitivity, spatial resolution, spectral sensitivity) but they can also be designed to provide important information about the underlying mechanisms of information processing. One area that has been explored behaviorally is how fish discriminate and/or categorize shapes. These studies have shown that fish can perform seemingly complex visual tasks such as image categorization (Schluessel et al., 2012), amodal completion (Sovrano and Bisazza, 2008), and perception of illusory contours (Wyzisk and Neumeyer, 2007). A range of species have been used in experiments including goldfish (Mackintosh and Sutherland, 1963; Bowman and Sutherland, 1969, 1970; Sutherland, 1969; Sutherland and Bowman, 1969; Douglas et al., 1988; Wyzisk and Neumeyer, 2007), redtail splitfin (Truppa et al., 2010), cichlids (Schluessel et al., 2012), damselfish (Mussi et al., 2005; Siebeck et al., 2009, 2010), groupers (Darmaillacq et al., 2011), parrotfish (Darmaillacq et al., 2011), weakly electric fish (Schuster and Amtsfeld, 2002; von der Emde et al., 2010), rays (Van-Eyk et al., 2011), and archerfish (Schuster et al., 2004; Schlegel et al., 2006; Segev et al., 2007; Ben-Simon et al., 2012b; Gabay et al., 2013; Newport et al., 2013; Rischawy and Schuster, 2013).

Archerfish are becoming increasingly popular as subjects for visual discrimination studies due in part to their unique hunting technique of knocking down insects in overhanging foliage using a jet of water. Several studies have focused on the mechanisms required for spitting (Milburn and Alexander, 1976; Waxman and McCleave, 1978; Elshoud and Koomen, 1985; Timmermans, 2000; Timmermans and Vossen, 2000; Timmermans, 2001; Rossel et al., 2002; Timmermans and Souren, 2004; Schuster et al., 2006; Schlegel and Schuster, 2008; Vailati et al., 2012) as well as their visual capabilities (Braekevelt, 1985a,b; Temple et al., 2010; Ben-Simon et al., 2012b; Temple et al., 2013). Recently a number of studies have also focused on the neural mechanisms of visual discrimination (Schuster et al., 2004; Schlegel et al., 2006; Segev et al., 2007; Ben-Simon et al., 2012a; Ben-Tov et al., 2013; Gabay et al., 2013; Rischawy and Schuster, 2013).

The goal of visual discrimination studies is to understand the circumstances under which a subject can perform relevant learning and discrimination, and beyond that, the robustness of the underlying representations to new exemplars of a target or to other objects within a category. In general terms, discrimination tasks in fish operate in a manner not unlike those conducted on human subjects. Visual stimuli are presented to the subject and some form of behavior is recorded as a response. Psychophysics tests can rely on observations of innate behaviors such as optomotor response or eye movements, as well as learned behaviors instantiated through classical and/or operant conditioning. Archerfish are particularly well suited for operant conditioning experiments as they are easily trainable, highly motivated, and their method of stimulus selection (i.e., hitting stimuli with a jet of water) produces an easily measurable response.

There are a number of psychophysical tests that can be employed to test the visual capabilities of fish (Schuster et al., 2011); however, a common approach is the two-alternative forced-choice (2-AFC) task. In this task, subjects are conditioned to associate a particular stimulus with a reward. The test involves identifying the conditioned stimulus (S+) when it is presented together with a single unconditioned distracter stimulus (S-). Archerfish have also been trained successfully to complete a 4-AFC task in which S+ is one of four stimuli (Ben-Simon et al., 2012b; Newport et al., 2013). While this test can be used to answer a wide range of questions about what an animal can discriminate, the conditioning process can be arduous as subjects have to be retrained to a new set of S+/S- stimuli following the completion of a particular experiment.

There are other psychophysical tests that do not require conditioning to particular stimuli but instead rely on the subject’s ability to learn associative rules such as the matched-to-sample (MTS) and odd-one-out (OOO) tasks. In the MTS task, the goal is for the subject to match a sample stimulus with a comparison stimulus (S+) shown in the presence of a distractor (S-). The sample can either be shown together with the comparison and distractor stimuli (simultaneous MTS), or the sample can be shown prior to the comparison and distractor stimuli being presented (delayed MTS). The delayed MTS can be used as a test of both working memory and visual discrimination ability. In a complementary method to the MTS, called the oddity-from-sample (OFS), the rewarded stimulus is the one which does not match the sample. Both reward systems require that subjects are able to discriminate the stimuli and to remember the sample.

The OOO task requires that subjects select a stimulus that is different amongst a set of like distracters. Unlike the delayed MTS/OFS paradigm, the OOO places only weak, if any, demands on working memory; subjects must simply discriminate between stimuli. However, crucially, in both types of task, subjects must learn the general concept of the task rather than simply associating a particular stimulus with a reward. Although conceptually more challenging, the advantage of the MTS/OFS and OOO tasks is that subjects do not need to be continually retrained to new stimuli. Not only can this decrease the time required to run an experiment, but also means that the discrimination capabilities of the subject can be tested with many stimuli, not just a particular conditioned one. It also makes it possible to reverse the role of test stimuli between target and distractor, reducing the chance that behavior is being driven by some inherent affinity the subject has for a particular visual feature or brightness level etc.

Knowing that archerfish can complete the MTS/OFS and OOO would be useful for the design of future discrimination experiments for several practical reasons, but may also provide insights into the cognitive abilities of these fish, namely their capacity for concept learning. Humans are notable in the animal kingdom for their extensive use of advanced concepts which are the foundation for the creation of language and numbers. Learning concepts can provide significant advantages to animals by allowing them to transfer previously gained knowledge to new objects and situations. As a result, it is reasonable to assume that these abilities did not arise solely in humans but have origins in other animals. Indeed, reports that animals can learn concepts (see Zentall et al., 2008, for a review of concept learning in animals) provide further evidence for this hypothesis. In humans the area of the brain associated with conceptual learning is the cortex (Martin, 2007; Binder and Desai, 2011). If fish, which lack a cortex, are unable to learn either of these tests it may suggest that they have trouble learning the associated concept and that the cortex is a requirement of higher learning. Likewise, if archerfish are able to learn the OOO task but not the delayed MTS/OFS task it may imply that they can learn concepts but do not have an adequate working memory. As a result, the inability of fish to perform a specific task may be just as telling as their ability to do it.

Most visual experiments involving fish have so far have used AFC tasks, however, Goldman and Shapiro (1979) and Zerbolio and Royalty (1983) did show that goldfish could complete a simultaneous MTS/OFS task. In a more recent study, experimenters were unable to train cichlids to complete a similar simultaneous MTS task (Gierszewski et al., 2013). It is important to note that all tests with fish have used a simultaneous MTS/OFS test where three stimuli were presented in each trial (the sample, S+ and S-). While subjects could solve the task by matching the sample with the comparison stimulus, it could also be solved by simply selecting or avoiding the stimulus that is different from the other two. As a result, it is impossible to determine if the fish had learned a matching task or an oddity task. We aimed to determine if archerfish could complete either or both of these tasks. To ensure that the fish were learning the MTS/OFS and not simply solving based on oddity, a delayed MTS/OFS was tested for the first time. As a comparison, the archerfish were additionally trained to complete a 4-AFC test. These results were used to evaluate how quickly archerfish could be retrained to new stimuli.

## MATERIALS AND METHODS

### SUBJECTS

Seven large-scale archerfish (*Toxotes chatareus*; Hamilton, 1822) were purchased from local suppliers. The total length ranged from 6 to 10 cm. All fish were kept in accordance with The University of Queensland Animal Ethics Committee approval (AEC Approval number: SBMS/241/12). Subjects were housed in individual aquaria (30 cm × 30cm × 60 cm) that served as both a holding and experimental tank. The fish were kept under a 12:12 h light: dark cycle using full spectrum fluorescent lights (F36T8/840, Cool White, Crompton, Australia) and supplied with recirculating fresh water maintained at 24 ± 0.5°C. Opaque dividers were placed between aquaria to ensure fish were unable to see each other, and therefore eliminate the possibility of observational learning. Fish were fed mini pellets (Cichlid Gold^®^, Kyorin Co. Ltd., Japan) daily as part of experiments. The fish had different levels of previous experience; however, all subjects had at least been pre-trained to spit at stimuli presented on a monitor, following methods described in Newport et al. (2013).

### APPARATUS

Stimuli were displayed on a 15 inch LCD monitor (SyncMaster 153v, Samsung) with a Plexiglas housing. This was suspended above the aquaria and oriented parallel to the water’s surface, as described in Newport et al. (2013). The stimuli were presented in different positions on the monitor depending on the experimental paradigm (see General Procedure). All stimuli were created using Microsoft PowerPoint and Adobe Photoshop CS5 (**Table 1**) and were 2.5 cm x 2.5 cm in size.

**Table 1:**
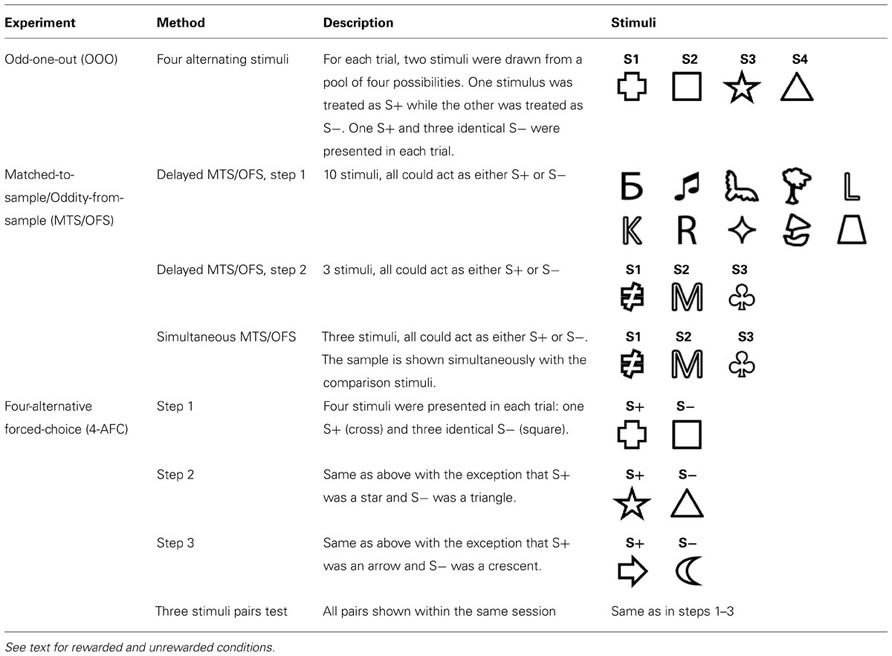
Summary of three experiments describing the stimuli used and brief experimental description.

### GENERAL PROCEDURE

Our aim was to test whether archerfish could learn the concepts required to solve OOO and MTS tests. A total of four experiments were conducted: (1) the OOO, (2) the delayed MTS/OFS, (3) the simultaneous MTS/OFS and (4) the 4-AFC. Different approaches can be used to train subjects and because neither the OOO nor MTS/OFS tests had been tested in archerfish before, the ideal training procedure was unknown. As a result, a series of training approaches was attempted so that if one method did not work, it would be possible to progress to a new one. A variety of simple line drawings (see **Table 1**), were used as stimuli in all experiments. These stimuli were chosen because Newport et al. (2013) showed that archerfish were able to easily discriminate these shapes. In our previous study, archerfish were trained to discriminate four shapes using a 4-AFC test (one S+ and three different S- stimuli). These results not only showed that archerfish use a variety of strategies when making decisions about stimuli but also that they are able to discriminate four trained shapes from 60 novel ones. Here, we also use shapes because they are easily discriminable by archerfish and therefore any breakdown in performance was more likely due to problems with the test itself and not the stimuli used. Methods for each experiment are described in detail below but see **Table 1** for a summary of all methods and stimuli used.

In all experiments, archerfish selected a stimulus by hitting it with a jet of water (referred to as ‘a hit’). The fish were rewarded with one food pellet each time they correctly hit S+. Incorrect choices terminated the trial without a reward and stimuli were removed from the monitor, except in some initial training sessions (the first 1–2 sessions) where the fish were given the opportunity to select various stimuli until they hit S+, at which point they were rewarded. This was to help the fish learn which stimulus was correct. In all following sessions the fish were only given one chance to make a selection. A squeegee was then used to remove water from the Perspex^®^ monitor cover. The next trial began after a brief delay. An individual was considered to have successfully learned the task once performance was significantly different from chance for two consecutive sessions (see Statistical Analysis for statistical calculations).

#### Odd-one-out

**FIGURE 1:**
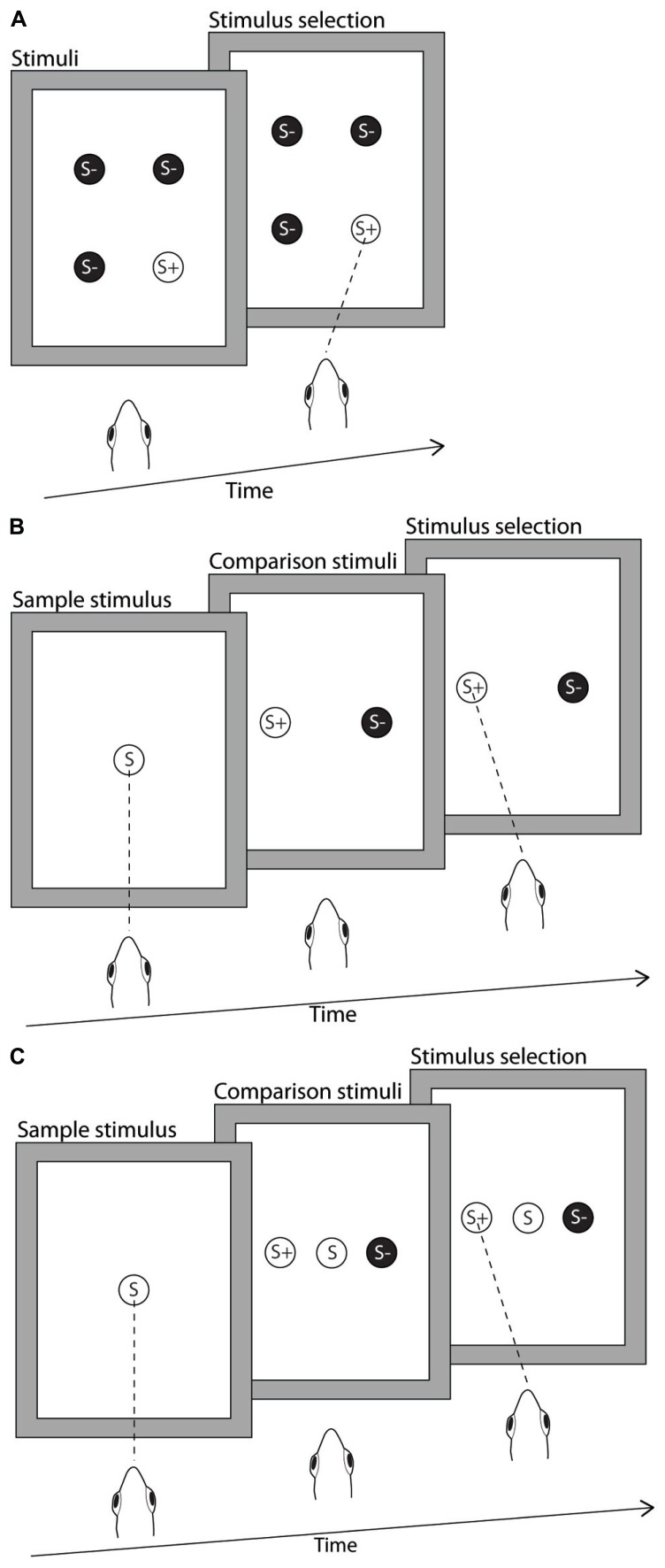
**Illustration of the stimulus presentation protocols used in the delayed and simultaneous matched-to-sample/oddity-from-sample (MTS/OFS), and the odd-one-out (OOO) task.** Stimuli were a range of black line drawings (not drawn to scale in figure) on a white background, presented on a computer monitor suspended directly above the aquarium. **(A)** Odd-one-out. The archerfish were presented with four stimuli, three identical S- and one different S+. These stimuli could appear in any of four possible positions on the monitor. The archerfish were required to select the single reward stimulus (S+). In this case the correct response is indicated as a dashed line representing a correctly aimed spit response. **(B)** Delayed MTS/OFS. The archerfish were presented with the sample stimulus in the middle of the monitor, shown here as S. The archerfish were required to hit the sample stimulus in order to trigger the display of the comparison stimuli and the removal of the sample. Of the two comparison stimuli, one stimulus was identical to the sample and the second stimulus was different from the sample. The fish was required to select the matching stimulus in the MTS test or select the different stimulus in the OFS test. In the figure, an example of a correct response is indicated as a spit to the reward stimulus. **(C)** Simultaneous matched-to-sample/oddity-from-sample. Similarly to the delayed MTS/OFS, a sample stimulus was presented in the middle of the monitor (S). However, once the archerfish hit the sample it remained on the monitor and the two comparison stimuli (S+ and S-) were immediately presented. The archerfish then selected either S+ or S- but selection of the sample stimulus was neither rewarded nor penalized.

Four fish (Fish 1, 2, 3, and 4) were trained to select the odd stimulus (S+) out of three other identical stimuli (S-). Four shapes (S1, S2, S3, and S4) were used as stimuli (**Table 1**) and all shapes could be both rewarded and unrewarded depending on whether they were acting as S+ or S-. In any given trial, only two of the four possible stimuli were presented, one being S+ and the other S-. There were four stimulus display positions on the monitor (monitor coordinates: -200 150, 200 150, -200 -150, and 200 -150) and the positions of all stimuli were randomized in all experiments with the constraint that S+ was never in the same position in consecutive trials (**Figure 1A**). Sessions were run until each subject completed 10 sessions (20 trials per session). If the subjects were able to successfully complete the task, two transfer sessions were run in which the four familiar shapes were exchanged for four novel stimuli. The transfer tests served to show if the fish had learned the concept of the OOO test in which case they should be able to transfer this knowledge to new stimuli.

#### Matched-to-sample/oddity-from-sample

***Delayed MTS/OFS.*** In the delayed MTS/OFS paradigm, the subject was first presented with a sample followed by a pair of comparison stimuli, one of which was identical to the sample. In the MTS task, the subject must select the comparison stimulus that matches the sample to receive a food reward. In the OFS task, the subject must select the stimulus that is different to the sample (**Figure 1B**). Two fish (Fish 3 and 6) were trained to the MTS task and a further two fish (Fish 5 and 7) were trained to the OFS task throughout all MTS and OFS experiments. The reason for training fish to complete the MTS and OFS task was that Newport et al. (2013) found that when archerfish learn a 4-AFC task S- plays an important role in learning and that the archerfish develop a strong association with S-. As a result, we hypothesized that archerfish may find the task easier if they were required to avoid the stimulus that matched the sample. Either approach provides a valid test of the fish’s ability to discriminate the two stimuli.

The training consisted of two steps. In step 1, 10 different shapes were used as stimuli (**Table 1**) and all shapes were used as both S+ and S-. A trial began when the sample stimulus was displayed in the center of the monitor (monitor coordinates: 0 0). Once the archerfish hit the sample, a key was hit by the experimenter which removed the stimulus from the monitor and caused S+ and S- to be presented on either side of where the sample stimulus had been shown (monitor coordinates: -90 0, 0 90). The positions of S+ and S- were randomized under the constraint that S+ was never in the same position in more than two consecutive trials and that S+ and S- were presented on each side equally often. The fish were rewarded with one food pellet every time they hit S+. Incorrect choices terminated the trial without a reward and stimuli were removed from the monitor. Between trials, a squeegee was used to remove water that had accumulated on the Perspex^®^ monitor cover. Daily training sessions consisted of 20 trials; except in rare cases where a fish would not complete every trial within a session due to variations in motivation. A total of 19 sessions was completed by all fish. In addition, the two fish that were trained to MTS were given an extra 10 pre-trials where only S+ was displayed after the sample. This was intended to reinforce the association between the sample and S+. The two fish trained to OFS were not given these pre-trials as it was impossible with this experimental design.

In step 2, the number of stimuli was reduced to three, all of which were used as both S+ and S-. These stimuli were different to those presented in the previous MTS/OFS procedures (**Table 1**). The number of stimuli was reduced because Goldman and Shapiro (1979) were successful at training goldfish to complete a simultaneous MTS task using only three stimuli. The procedures were identical to those of MTS/OFS methods 1. Sessions consisted of 20 trials and 10 sessions were completed.

***Simultaneous MTS/OFS.*** The methods used by Goldman and Shapiro (1979) were replicated to train the archerfish to complete a simultaneous MTS/OFS task. The difference between the simultaneous and delayed MTS/OFS is that the sample remains in place and the two stimuli choices (S+ and S-) appear on either side of the sample once the archerfish hits the sample (**Figure 1C**). In this situation there is no consequence to hitting the sample (it is neither rewarded nor causes the termination of a trial) so this is still considered a two choice test and selection frequency is expected to be 50% if at chance. All other components of the procedure were the same as in the delayed MTS/OFS step 2 including the stimuli used. A total of 40 sessions was attempted for all fish; however, due to variations in motivation not all fish completed the full 40 sessions.

At the conclusion of the simultaneous MTS experiment, a control test was run to determine if the archerfish could discriminate the three shapes. This was done in order to eliminate the possibility that the archerfish were unable to complete this task due to a breakdown in discrimination ability. Two fish (5 and 7) were presented with a 3-AFC task and were trained to select one S+ from two different S-. Each fish was trained to a different S+ to ensure that an individual S+ was not affecting performance. Stimuli were presented in the same positions as described for all MTS tasks. Fish 3 and 6 did not complete this control test due to a lack of motivation to participate in any further testing. Fish 5 was trained to select S1 and Fish 7 was trained to select S2 (**Table 1**).

#### Four-alternative forced-choice

The archerfish were trained to complete a 4-AFC test in which four stimuli were presented in each trial (one S+ and three identical S-). To determine how many sessions were required to retrain the fish to novel stimuli, the fish were then conditioned to two novel stimuli. A further test was run with another two novel stimuli to determine if retraining to new stimuli required less sessions when the fish had practice. Finally, a test was run with all three conditioned pairs in the same session. This was done to determine if archerfish could remember up to three conditioned stimulus pairs at the same time which may allow for greater flexibility for the design of future experiments.

Four fish (Fish 1, 2, 3, and 4) were conditioned to discriminate between one cross (S+) and three identical squares (S-). There were four stimulus display positions on the monitor (monitor coordinates: -200 150, 200 150, -200 -150, and 200 -150) and the positions of all stimuli were randomized in all experiments with the constraint that S+ was never in the same position in consecutive trials. Sessions consisted of 20 trials and were run until each subject had completed a minimum training criterion of five sessions and reached an S+ selection frequency ≥70% in two consecutive sessions. This criterion was chosen because in order for a task to be used as a visual discrimination test, subjects should be able to complete each training task with a high degree of accuracy and should demonstrate consistency in their performance. This is to ensure that when analyzing performance during transfer tests with new stimuli, any changes in fish behavior are due to the new stimuli and not simply stochastic variation. Archerfish have been shown to reach accuracy levels of up to 95% when presented with a 4-AFC test with shapes as stimuli (Newport et al., 2013) which is much higher than required for significance. 

The stimuli were then substituted for a second pair; a triangle (S+) and three identical stars (S-) and the same method was repeated as described above. After each fish had completed the required training sessions, a third pair of stimuli was introduced: an arrow (S+) and three identical crescents (S-). Once the fish had learned all three stimulus pairs, a test was run to determine if the fish could continue to complete the task when all three pairs were presented within the same session. For each trial, one pair was chosen at random with the restriction that the same pair was not shown in two consecutive trials and all pairs were shown equally often. Two test sessions were run. See **Table 1** for stimuli.

### STATISTICAL ANALYSIS

Selection frequencies for each stimulus type (S+ or S-) were calculated for each condition per fish by tallying the number of hits for all trials per session. The raw data were analyzed using a Chi-square test. In both the AFC and OOO paradigms four stimuli are presented. As a result, the expected selection frequency of S+ if chosen at random is 25%. A selection frequency of S + ≥ 45% (*n* = 20 trials) is statistically significant (*P* = 0.039). In the MTS/OFS task, only two stimuli can be chosen so the expected selection frequency of either stimulus is 50% if chosen at random. A selection frequency ≥75% (*n* = 20 trials) is statistically significant (*P* = 0.025).

A Chi-square test was used to test for positional bias. For the AFC test, the two test sessions were tested for positional bias and for the OOO and MTS/OFS tests the last two sessions completed by the subject were tested (*n* = 40 trials). The expected selection frequency of each position is 25% in the AFC and OOO tests and 50% in the MTS/OFS tests. An additional test of the same sessions was done for stimulus selection bias using a Chi-square test. In both the AFC and OOO procedures, not all stimuli are presented within a trial, however, the presentation of each stimulus is balanced so that all stimuli are shown in equal frequencies within a session. Therefore, the expected selection frequency of each stimulus is 16.6% in the AFC test (six different stimuli) and 25% in the OOO test (four different stimuli). For MTS/OFS tests the expected S+ selection frequency with 10 stimuli is 10% and 33.3% with three stimuli. Only the last two sessions were used because training is a learning process and as a result we only wanted to test the sessions where the fish was most likely exhibiting the learned behavior.

## RESULTS

### ODD-ONE-OUT

**FIGURE 2:**
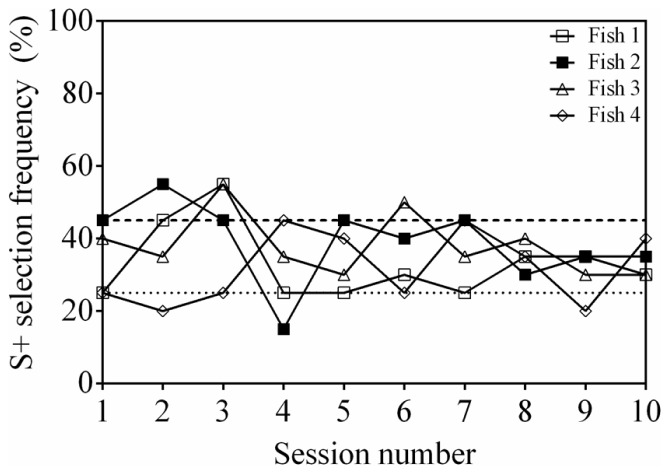
**Discrimination performance as a function of time (binned by testing session), for four fish performing an odd-one-out task.** Two stimuli were selected for each trial from a pool of four possibilities. See **Table 1** for stimuli used. The dashed line at 45% indicates a statistically significant selection frequency of S+ and the dashed line at 25% indicates chance.

Of the four fish tested, two individuals (Fish 1 and 2) were able to reach a significant selection frequency (S+ selection ≥45%) in two consecutive sessions (**Figure 2**). However, the accuracy of these subjects was variable and selection of S+ was significant in only some of the 10 sessions (two and five sessions, respectively). Two fish (Fish 3 and 4) were also able to reach an accuracy above chance, however, performance was again inconsistent and significance was only achieved in two sessions out of 10 each. Because Fish 2 reached significance in three sessions, two transfer tests with new stimuli were completed, however, only an S+ selection frequency of 20% in the first session and 35% in the second session was achieved, which are not significantly different from chance (session 1: *P* = 0.606; session 2: *P* = 0.302).

A test for positional and stimulus bias was run for all fish. Fish 2 was the only individual to exhibit a positional bias (*P* < 0.001), predominantly selecting stimuli in position 1 (position 1: 50%; position 2: 27.5%; position 3: 15%; position 4: 7.5%). This individual was also the only one to exhibit a significant stimulus bias (*P* < 0.05) selecting S4 in 45% of trials (S1: 32.5%; S2: 17.5%; S3: 5%).

### MATCHED-TO-SAMPLE/ODDITY-FROM-SAMPLE

#### Delayed MTS/OFS

**FIGURE 3:**
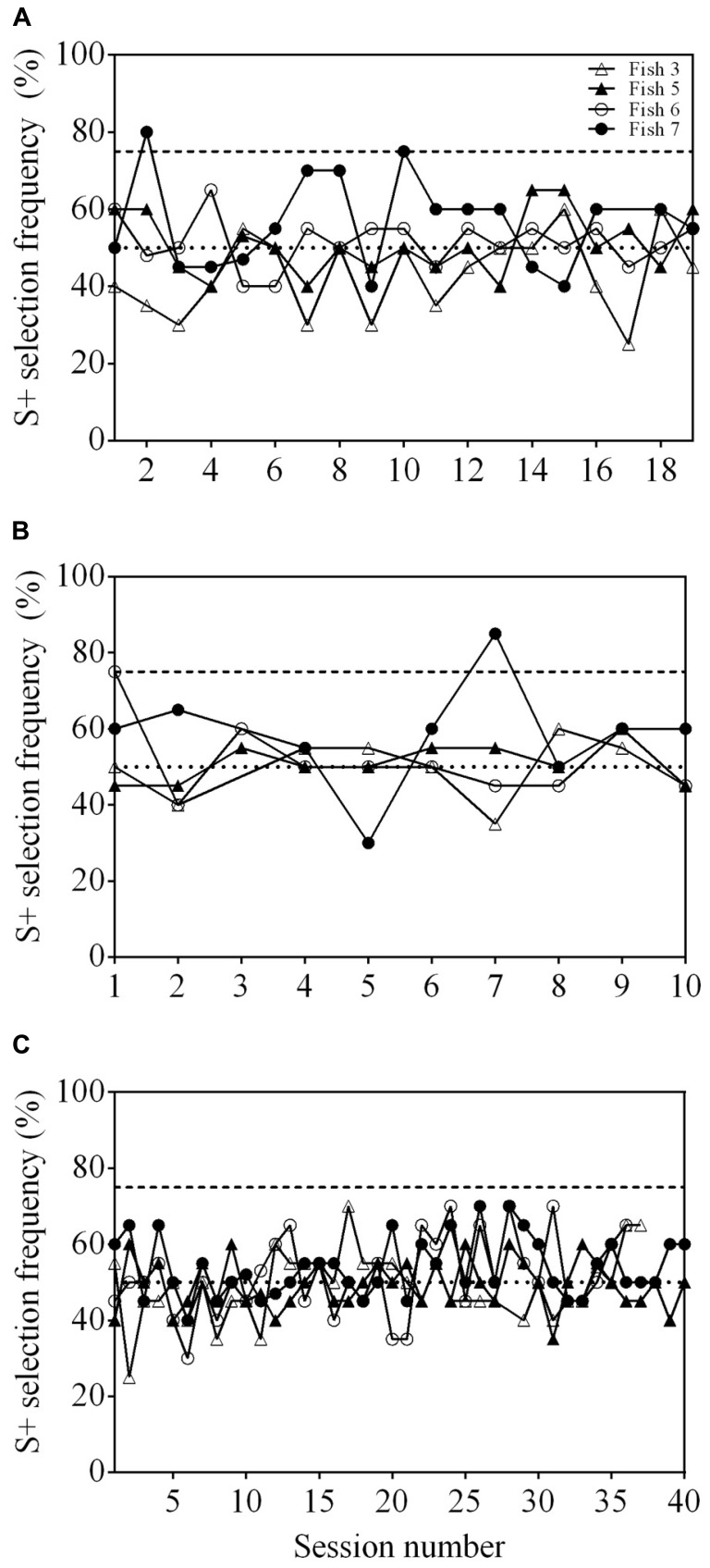
**Results of three training procedures for matched-to-sample/oddity-from-sample (MTS/OFS) tasks. (A)** Learning curve of four archerfish when presented with a delayed MTS/OFS task. A pool of 10 shapes was used as stimuli. **(B)** Learning curve of four archerfish given a similar delayed MTS/OFS task with the modification that the pool of stimuli used was reduced to three. **(C)** Learning curve of four archerfish given a simultaneous MTS/OFS task using a pool of three shapes as stimuli. The dashed line at 75% in all figures indicates a statistically significant selection frequency of S+ and the dashed line at 50% indicates chance. Filled symbols represent fish trained to an OFS task and empty symbols represent fish trained to a MTS task. See **Table 1** for example stimuli.

Neither Fish 3, 5, nor 6 was able to reach statistical significance after 19 sessions in step 1 (**Figure 3A**). Fish 7 did achieve an S+ selection frequency ≥75% in two out of 19 sessions, however, never in consecutive sessions.

The final two sessions of step 1 for each fish were tested for a possible positional bias. Three of the fish (Fish 3, 5, and 6) exhibited a significant side bias (*P* < 0.001). While Fish 3 selected stimuli on the right side at a higher frequency, Fish 5 and 6 preferred stimuli on the left. Fish 7 showed no preference for either stimulus position (*P* = 0.114). None of the fish showed a preference for any of the 10 stimuli presented (Fish 3: *P* = 0.689; Fish 5: *P* = 0.941; Fish 6: *P* = 0.834; Fish 7: *P* = 0.534).

Following the delayed MTS/OFS task with 10 stimuli, a further 10 sessions were completed in which the number of stimuli presented was reduced to three. Two individuals, Fish 6 and 7, reached significance for one session each, however, the other two fish (Fish 3 and 5) did not (**Figure 3B**).

**FIGURE 4:**
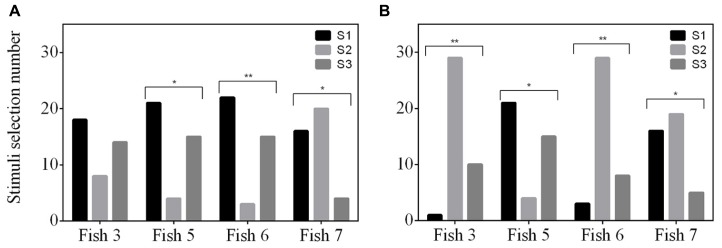
**Selection frequency of three stimuli (S1, S2, and S3) for two experiments; (A) delayed MTS/ OFS, and (B) simultaneous MTS/OFS.** See **Table 1** for example stimuli. The total trial number was 40 for each experiment and the selection frequency of each stimulus was tested for a selection preference using a Chi-square test.

No fish exhibited a positional bias in the final two sessions (Fish 3: *P* = 0.527; Fish 5: *P* = 0.206; Fish 6: *P* = 0.527; Fish 7: *P* = 0.342) however, three of the fish did show a stimulus bias (Fish 3: *P* = 0.149; Fish 5: *P* = 3.74 × 10^-^^3^; Fish 6: *P* = 9.66 × 10^-^^4^; Fish 7: *P* = 5.44 × 10^-^^3^). All three fish avoided one of the stimuli, however, the stimulus avoided varied between fish (**Figure 4A**).

#### Simultaneous MTS/OFS

None of the fish were able to achieve an S+ selection frequency ≥75% (**Figure 3C**). The number of sessions completed was variable between fish and, as a result, Fish 6 only completed 36 sessions and Fish 3 completed 37. Both Fish 5 and 7 completed all 40 sessions. No fish exhibited a position bias in the final two sessions (Fish 3: *P* = 0.527; Fish 5: *P* = 0.107; Fish 6: *P* = 0.527; Fish 7: *P* = 0.342). All fish had a significant stimulus selection bias and avoided one of the stimuli; however, the stimulus avoided varied between fish (**Figure 4B**).

**FIGURE 5:**
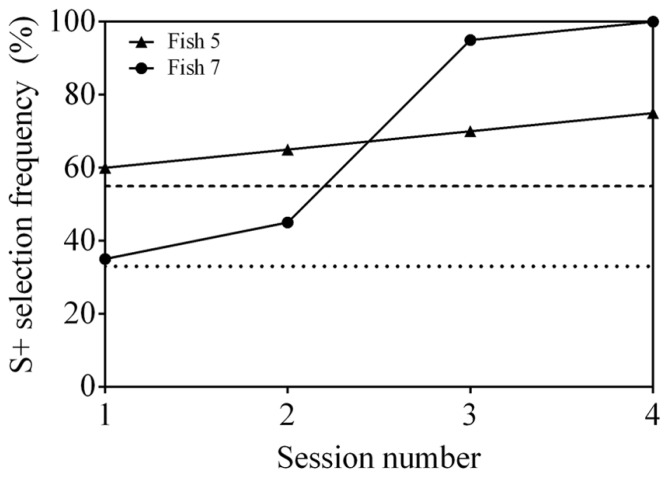
**Discrimination performance as a function of time (binned by testing session), indicating the steady improvement observed for two fish carrying out the 3-AFC task.** Fish 5 and 7 were trained to select S1 and S2, respectively (**Table 1**).

Both fish 5 and 7 were able to successfully learn the 3-AFC control test and reached a statistically significant S+ selection frequency (≥55%) in two consecutive sessions within four sessions (**Figure 5**). These results indicate that the stimuli used could be discriminated by the fish.

### FOUR-ALTERNATIVE FORCED-CHOICE

**FIGURE 6:**
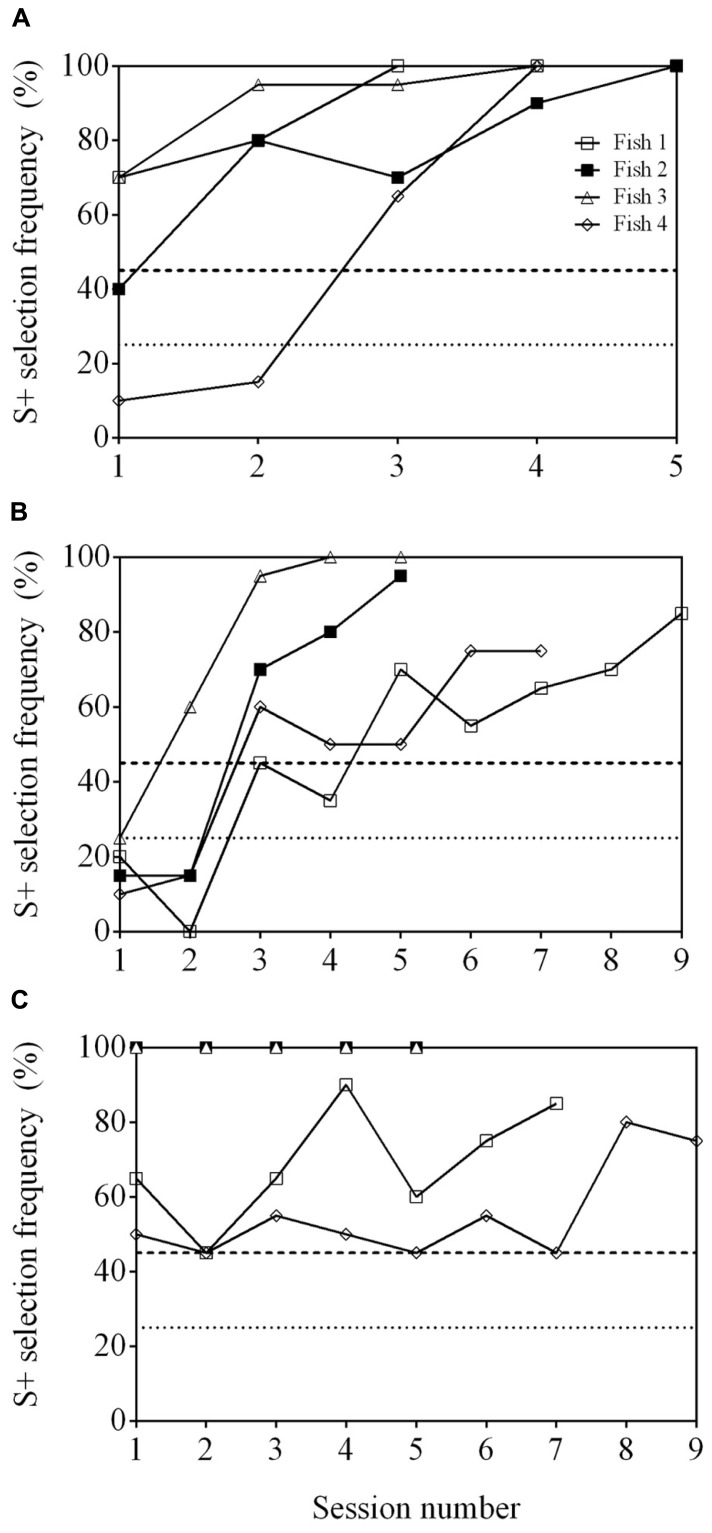
**Learning curve of four archerfish conditioned to complete a 4-AFC task.** Four stimuli were presented where three stimuli were identical and were unrewarded (S-) while a single unique stimulus was rewarded (S+). In **A**, S+ is a cross and S- is a square. The stimuli were then replaced by a star (S+) and a triangle (S-; **B**). Stimuli were changed for a third time to an arrow (S+) and a crescent (S-; **C**). The dashed line at 45% in all figures indicates a statistically significant selection frequency of S+ and the dashed line at 25% indicates chance. See **Table 1** for example stimuli.

**FIGURE 7:**
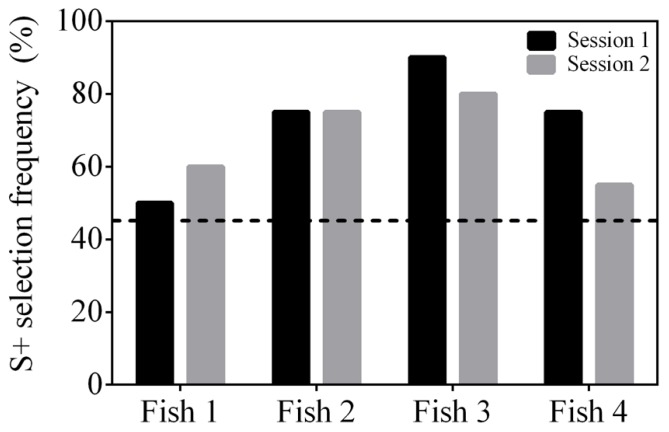
**Selection frequency (%) of S+ using a 4-AFC test where all three conditioned stimulus pairs were presented within a session.** The results of two testing sessions (*n* = 20 trails each) are presented for four subjects. The dashed line at 45% indicates a statistically significant selection frequency of S+. All subjects achieved an S+ selection frequency above chance.

All fish were able to reach well above a statistically significant S+ selection frequency (≥45%) when presented with a cross (S+) and a square (S-) within 2–3 sessions (**Figure 6A**). They continued to reach ≥45% when presented with the second stimulus pair, a triangle (S+) and star (S-), but took 4–9 sessions to do so (**Figure 6B**). The final stimulus pair was an arrow (S+) and a crescent (S-). All fish reached ≥45% within 2–9 sessions (**Figure 6C**) and two of the fish (Fish 2 and 3) achieved 100% accuracy in all five sessions. Regardless of the stimuli presented, the fish were able to be re-trained and complete the task with different stimuli. All fish were able to select S+ at a frequency ≥45% when all three stimulus pairs were presented within the same session showing that they could complete the task (**Figure 7**).

## DISCUSSION

The overall aim of this project was to explore the ability of archerfish to solve two concept based psychophysics tests. The MTS/OFS and the OOO tests both require that the subject learn a concept rather than simply learning to associate a particular stimulus with a reward. One benefit of these tests is that a large number of stimuli can be tested within a single experiment without having to continuously retrain the subject to new stimuli. Another benefit is that they can provide information about how subjects are able to learn to complete complex tasks. The results of the OOO test show that two out of four archerfish reached a statistically significant S+ selection accuracy in two consecutive sessions and therefore passed the test. In contrast, none of the four archerfish were able to reach statistical significance in two consecutive sessions in the delayed or simultaneous MTS/OFS test. Our findings indicate that some archerfish may be able to learn the concept based OOO tests, however all were unable to learn the MTS/OFS regardless of the training procedure used. A 4-AFC test was then conducted as a comparison to the other tests and to assess how easily archerfish could be retrained to new stimuli. All archerfish reached a much higher S+ selection accuracy in the 4-AFC test with one S+ and three identical S- (present study) and the 4-AFC in which all four stimuli were different (Newport et al., 2013) than in the MTS/OFS or the OOO tests. We found that retraining archerfish to new stimuli required few sessions and that they could be trained to recognize up to three conditioned stimulus groups at once. In addition, we found after training the fish to two different sets of stimuli, some individuals were able to achieve 100% accuracy within the first training session with new stimuli pairs. This would appear to indicate that archerfish are capable of generalizing their learning to novel stimuli, indicative of some degree of task relevant conceptual learning, rather than merely stimulus specific learning.

The OOO test requires that subjects apply the concept of oddity to solve the task. It has been primarily used as a test for visual discrimination in primates but has been shown to be solvable by other animals such as pigeons (Blough, 1986), cats (Boyd and Warren, 1957), and goats (Roitberg and Franz, 2004). It has never before been tested in fish. In this test, each archerfish was given 10 training sessions (200 trials). The results of our experiments show that all four archerfish were able to reach statistical significance in a combined 11 out of 40 sessions (2, 5, 2, and 2 sessions, respectively) yet only two of these fish (Fish 1 and 2) could do this in consecutive sessions. These results suggest that two of the fish had learned the task. The probability of reaching our learning criteria by chance in a particular session, and thereby getting a false positive result, is *P* = 0.0389 (*n* = 20 trials). Therefore within the 10 sessions performed by four fish, we would expect two sessions to be positive due to chance (0.0389*10*4 = 1.55). Therefore it is unlikely that our observed results are simply due to false positives. It is even less likely considering that two of the fish reached an S+ selection accuracy of ≥45% in consecutive sessions. However, there appears to be no learning curve whereby performance improves over the number of training sessions. In addition, when Fish 2 was given a transfer test in which the stimuli were changed for novel shapes, performance was at chance. True evidence that the concept of oddity has been learned requires that the subject apply the concept to novel stimuli. As a result, it appears as though the archerfish may have had only a limited understanding of this task if at all. This is somewhat surprising as this task is likely to be of ecological relevance to many species of fish. For example, targeting rare prey in a group increases the chance of predatory fish catching their prey (Landeau and Terborgh, 1986; Theodorakis, 1989; Almany et al., 2007). However, it is possible that archerfish gain no such advantage in singling out a rare object and have therefore not developed this skill. Archerfish are generalist feeders that encounter many insect species in their natural environment. In order to catch insects, they must spit at many potential food sources and only make a decision about whether or not to ingest something after they have taken it into their mouth and “tasted” it. As a result, visually selecting an individual insect from a crowd may not provide any benefit to archerfish. It may be possible that other species, especially predators that hunt schooling fish, will prove more adept at the OOO task. Future experiments are required to test this hypothesis.

In the MTS/OFS test, subjects must apply the concept of matching to select or avoid a stimulus that is the same as a previously presented sample stimulus. A series of training procedures was attempted to train the archerfish to the MTS/OFS test; however, the results of all three MTS/OFS training procedures show similarities in that all fish were unable to perform the task in more than one consecutive session. In step 1, all fish were allowed 19 sessions (380 trials) and in step 2, all fish were given a further 10 sessions (200 trials). In the simultaneous MTS/OFS two fish completed 40 sessions (800 trials) while one fish completed 36 (720 trials) and another completed 37 (740 trials) sessions. Although two (Fish 6 and 7) fish did reach above significance on occasion, these match the number of expected false positives. As was observed in the OOO test, there was no evidence of improved performance throughout the training period. The archerfish showed similar results in both the delayed and simultaneous MTS, making it unlikely that their poor performance was due to a lack of working memory alone. In addition, Newport et al. (2013) found evidence that when solving a task where multiple stimuli are presented, archerfish examined each stimulus individually, a behavior which would require some form of working memory. It is more likely that the archerfish lacked the ability to understand the relationship between the sample and the comparison stimuli and, as a result, did not learn the concept of “sameness/difference.” Primates can learn this “sameness/difference” concept (e.g., Premack, 1976; Oden et al., 1988; Fagot et al., 2001; Wasserman et al., 2001; Young and Wasserman, 2001, 2002) and there is evidence that non-primate species such as bees (Giurfa et al., 2001), dolphins (Herman et al., 1989, 1994; Mercado et al., 2000), sea lions (Pack et al., 1991; Kastak and Schusterman, 1994) and pigeons (e.g., Blaisdell and Cook, 2005; Bodily et al., 2008) are also capable of doing so. Based on our results and those of Goldman and Shapiro (1979), Zerbolio and Royalty (1983) and Gierszewski et al. (2013) it appears as though the answer for fish may be dependent on the species and possibly their particular ecology.

Archerfish were then trained to complete a 4-AFC test. Although the 4-AFC test has been proven to provide reliable results (Ben-Simon et al., 2012b; Newport et al., 2013), it is limited by the fact that subjects must be conditioned to a particular stimulus. It was thought that retraining fish to new stimuli would take just as many sessions as initial training, but this had not yet been shown experimentally. Following the initial training, the archerfish were trained to two additional stimulus pairs. We found that the archerfish generally learned new S+/S- combinations in fewer sessions in step 3 than required for initial training. In the initial training test and the first test with new stimuli, all fish showed typical learning curves where accuracy generally increased as more sessions were completed. However, when the stimuli were changed for a third time, two fish were able achieve an accuracy of 100% within the first session. In a 4-AFC test where all distractors are the same, it is possible to solve the task by simply applying the concept that the one stimulus that is different is the correct answer. The ability of some individuals to solve the task immediately suggests that the fish learned the concept of selecting the single S+ stimulus and could apply it to new stimuli. What is different between the OOO and 4-AFC test is that the role of the stimuli did not change in the 4-AFC test. In the OOO test the same stimuli could be used as both S+ and S- whereas in the AFC a particular stimulus could only represent either S+ or S-. For archerfish, the concept of oddity may break down once the same stimuli are used as both S+ and S-. It is possible that reassigning the role of a learned object is unnatural for archerfish. For example, if the fish had learned that an object had a negative association (i.e., it was unrewarded or inedible), it may be rare that the properties of that object would change to being positive (i.e., the object becoming more palatable). As a result, once archerfish learn the role of an object they do not easily reverse their association.

Not all fish applied this strategy and instead exhibited a similar learning curve as observed in the previous two experiments except that they selected S+ at a frequency higher than chance within the first session. The number of sessions required to learn each task was variable. In all tests, Fish 1 consistently required more sessions to learn than the other three fish. It is possible that this fish did not understand the task as easily as the others. Alternatively, archerfish individuals have been shown to apply different decision strategies when solving the AFC test (Newport et al., 2013). It is possible that Fish 1 was using a different strategy from the other fish that required more sessions to learn. A third alternative is that this individual had a different level of motivation for completing the task. A final test was completed in which the fish were faced with all three pairs of stimuli within the same session. This was done to determine if they could remember multiple conditioned stimuli at the same time. All four fish were able to complete this task. Although using new stimuli does require retraining, our results show that fish can progressively learn faster and faster. In addition, they can learn more than one set of stimuli at a time, meaning that more complex experiments can be designed.

It is interesting to note that when the archerfish did not grasp the MTS/OFS or OOO tasks, they did not simply choose stimuli at random but instead resorted to using at least two different strategies to solve the problem. When confronted with a difficult task it is common for fish to develop a strong preference for stimuli on a particular side (Northmore and Yager, 1975). In the case of the delayed MTS/OFS test where 10 different stimuli were used, three of the four fish tested, developed a side bias. In experiments where fewer stimuli were used such as the OOO test with four alternating stimuli, the simultaneous MTS/OFS and the delayed MTS/OFS with three stimuli, the fish generally developed a stimulus bias in which they had a hierarchal preference for stimuli.

The results of our experiments provide some interesting insight into the limitations of the fish brain. Because of the nature of the tests used, the poor performance of the archerfish when presented with the MTS/OFS and OOO tests could suggest a deficiency of the working memory or an inability to learn concepts. Newport et al. (2013) found evidence from the 4-AFC test that archerfish consider stimuli independently and sequentially based on the fact that the anatomy of their eye makes it unlikely they could view more than one stimulus at a time and the fact that there were variable reaction times when responding to different stimulus types. This indicates that archerfish have an adequate working memory to consider all stimuli on the monitor and therefore to at least perform the simultaneous MTS and OOO tests. The problem then may lie with concept learning. Traditionally it has been thought that the evolution of vertebrate brains has progressed linearly in increasing complexity. Fish, the most primitive vertebrate group, therefore would have the simplest brains and would be expected to be incapable of more complex tasks. However, there is increasing evidence that fish share similar learning and memory capabilities with other vertebrates and that these are based on equivalent or similar neural mechanisms and brain systems. For example, classical conditioning of simple motor responses such as eye blink responses occurs in the cerebellum in both mammals (Thompson and Steinmetz, 2009) and fish (Gómez et al., 2010). Similarly, emotional conditioning and spatial memory is linked to the telencephalon and cerebellum of fish and homologous structures such as the amygdala and cerebellum of mammals (see Broglio et al., 2011, for a review of the neural mechanisms of cognition in fish). In humans, the frontal cortex is generally associated with abstract rule learning (Strange et al., 2001; Koechlin et al., 2003; Bunge, 2004; Bor and Owen, 2007; Christoff and Keramatian, 2007) and therefore it is possible that since fish lack a cortex, they will be unable to learn concepts. However, the neural mechanisms of concept learning in fish have not yet been examined and it is impossible to say if fish have homologous structures that enable them to perform this task. The results of the AFC retraining described in this report suggest that archerfish are capable of learning some sort of relational concept and predatory fish are able to apply the concept of oddity to hunting prey (Landeau and Terborgh, 1986; Theodorakis, 1989; Almany et al., 2007). In addition, other animals lacking a cortex are capable of the concept based MTS/OFS (bees: Giurfa et al., 2001; birds: Zentall and Hogan, 1974; goldfish: Goldman and Shapiro, 1979; Zerbolio and Royalty, 1983) and OOO (birds: Blough, 1986) tasks. The fact that both archerfish and cichlids (Gierszewski et al., 2013) appear incapable of learning the MTS/OFS task yet goldfish can, suggests that fish in general may have the neural mechanisms required for concept learning; however, different species may apply different decision rules which limit their performance. The ability to complete this task may come down to the general ecology of the species. Alternatively, it is possible that some species have evolved specialist hardware for this sort of task. Of course we cannot exclude the possibility that our training procedures did not adequately convey the task to the archerfish. Although we tried a range of training procedures, it is possible that different training techniques may elicit better performance. The combined evidence from fish, birds and bees, all of which lack a cortex, suggests that having a cortex is not a requirement for learning abstract relationships and concepts. However, many of these tests show that these animals can have limitations in their capabilities such as decreased performance when novel stimuli are introduced (Zentall and Hogan, 1974; Giurfa et al., 2001). It may be that a lack of cortex limits the flexibility of learning these concepts and that comprehension can only occur under specific conditions. However, one should be cautious in over-interpreting our results and more focused research in this field is required.

Although our results suggest that archerfish are incapable of learning the MTS/OFS and OOO tests, it is possible that they would be able to learn these under different experimental conditions. In our experiments we used a range of shapes as stimuli as previous studies have shown that archerfish are capable of discriminating a large number of shapes from four trained shapes (Newport et al., 2013). Shapes are a common stimulus class for behavioral studies and have previously been used in successful concept learning studies (e.g., Herman et al., 1989; Pack et al., 1991; Bodily et al., 2008), however, other studies have employed different stimuli such as colors (e.g., Goldman and Shapiro, 1979; Giurfa et al., 2001) and patterns (Giurfa et al., 2001). It is possible that although archerfish can discriminate shape stimuli, they may not be able to learn the concept of similarity based on this stimulus class. As a result, the use of different stimulus classes may yield different results. Pilot studies were run for the OOO test in which three different stimulus classes were tested: colors (red, blue, yellow, and gray), directional arrows and shapes, however, no difference in performance was found. When training animals, it can sometimes be difficult to successfully communicate the task, especially when trying to convey an abstract concept. Subtle changes in procedure can have an impact on the ability of the subject to understand the task. As a result, a range of training methods were attempted during pilot studies. For example, the feedback to errors in stimulus selection was varied in an attempt to make the consequences greater (i.e., a tone was played if the choice was incorrect or a timeout of 30 s was introduced before a new trial could commence). However, the methods described in this manuscript were those that were found to engender the most success when training archerfish to complete an AFC test. Future attempts to test concept learning in archerfish would likely have the most chance of success if they focused on changes in how the stimuli are presented. For example, in the OOO test described in this report four stimuli were presented, one of which the fish had to choose. Future experiments may be more successful if a much larger number of distractor stimuli were presented.

Another consideration is the duration of training. As this was the first time these paradigms have been attempted in archerfish, it is difficult to know how much training might be required. Evidence from other animals can be difficult to use as a guide as a range of factors can influence how many trials and sessions can be completed. For example, the number of trials that an animal can complete per session is highly variable. While animals such as baboons (Fagot et al., 2001) and pigeons (Bodily et al., 2008) can readily complete 96 trials per session, dolphins (Herman et al., 1989), and sea lions (Pack et al., 1991) typically only do between 8 and 28 trials. In behavioral experiments involving fish, they are commonly given between 6 and 10 trials (e.g., Siebeck et al., 2009, 2010; Truppa et al., 2010; Schluessel et al., 2012; Gierszewski et al., 2013), however, goldfish are capable of completing 100–120 (e.g., Goldman and Shapiro, 1979; Wyzisk and Neumeyer, 2007). Although archerfish have the motivation to complete a large number of trials in one session, we found during pilot experiments that archerfish performed best over long periods if given 20 trials per session. Because of the large variation in trial number that can be performed, it is difficult to compare the total number of trials required to learn a task between species. It is not known how much trial number affects the performance of fish and it is possible that the number of sessions is more relevant. Session number can also be difficult to use as a guideline because of the large discrepancies amongst different species. For example, pigeons were able to learn a MTS task within 11 sessions (Bodily et al., 2008) while bees and dolphins required 6 (Herman et al., 1989; Giurfa et al., 2001) and sea lions required 36 (Pack et al., 1991). Goldman and Shapiro (1979) reported that goldfish learned the simultaneous MTS and oddity-from-sample within 11–60 sessions; however, most individuals showed signs of improvement within the first 10 sessions. In this report as well as Gierszewski et al. (2013), a total of 40 sessions was attempted for the simultaneous MTS/OFS after the fish had already completed a total of 29 sessions for step 1 and 2 of the delayed MTS/OFS task. While it is possible that archerfish could eventually learn with more trials and sessions, we decided that any more than this would make the test impractical as a visual discrimination testing paradigm and therefore did not continue. In the case of the OOO, fewer sessions were completed. Despite the large number of sessions conducted in the combined MTS/OFS tests there was no improvement in performance with an increasing number of sessions and therefore we found it unlikely that conducting large numbers of sessions would improve our results. We found that in the MTS/OFS experiments, the archerfish eventually lost motivation and after about the first 10 sessions rarely changed their decision strategy (i.e., side or stimulus bias). In addition, in the simultaneous MTS/OFS experiments with goldfish, a large number of sessions were required for some individuals to reach significance; however, they at least showed some improvement within 10 sessions. In the case of the archerfish, no learning curve was observed whereby accuracy improved over time. For the purpose of identifying other paradigms that maybe useful for future testing, completing more trials and sessions is impractical; however, future studies focused on concept learning in general may want to attempt more sessions. If that is the case, it may be useful to change the food reward to be smaller or less nutritious or to use an intermittent reward schedule.

Our results indicate that archerfish were unable to learn the MTS/OFS task and only a few individuals were able to significantly select S+ in the OOO task but showed inconsistent performance. Although it is possible that archerfish may be able to learn concepts under different experimental conditions, we conclude that both of these tests are poor choices for visual discrimination experiments involving archerfish. However, our results indicate that archerfish achieve a very high accuracy when completing a 4-AFC test and can be rapidly retrained to new stimuli. In a 4-AFC test in which the three S- stimuli are identical, archerfish can learn to select the single S+ stimulus and therefore require no retraining when new stimuli are presented. The ability of archerfish to select odd stimuli can be used in a similar way to a traditional OOO test, in which subjects learn to select the singleton stimulus, with the limitation that stimuli are not presented in the role of both S+ and S-. This report not only provides important insight into concept learning in fish but also provides a powerful new technique that can be added to the tool box of psychophysical experiments used to explore vision in fish.

## Conflict of Interest Statement

The authors declare that the research was conducted in the absence of any commercial or financial relationships that could be construed as a potential conflict of interest.
